# Infiltration of T Cells into a Three-Dimensional Psoriatic Skin Model Mimics Pathological Key Features

**DOI:** 10.3390/ijms20071670

**Published:** 2019-04-03

**Authors:** Isabelle Lorthois, Mélissa Simard, Sophie Morin, Roxane Pouliot

**Affiliations:** 1 Centre de recherche en Organogénèse Expérimentale de l’Université Laval/LOEX, Axe Médecine Régénératrice, Centre de Recherche du CHU de Québec, Université Laval, Québec, QC G1J 1Z4, Canada; isabelle.lorthois.1@ulaval.ca (I.L.); melissa.simard.6@ulaval.ca (M.S.); sophie.morin.7@ulaval.ca (S.M.); 2 Faculté de Pharmacie, Université Laval, Québec, QC G1V 0A6, Canada

**Keywords:** psoriasis, T cells, adaptive immunity, inflammation, 3D model, tissue engineering

## Abstract

Psoriasis is an autoimmune chronic dermatosis that is T cell-mediated, characterized by epidermal thickening, aberrant epidermal differentiation and inflammatory infiltrates, with a dominant Th1 and Th17 profile. Additional in vitro models are required to study the complex interactions between activated T cells and skin cells, and to develop new, more effective treatments. We have therefore sought to model this psoriatic inflammation by the generation of tissue-engineered immunocompetent tissues, and we have investigated the response of activated T-cell infiltration in models produced with lesional psoriatic skin cells on major hallmarks of psoriasis. The immunocompetent lesional skin model displayed a delayed onset of epidermal differentiation, an hyperproliferation of the basal keratinocytes, a drastic increase in the secretion of proinflammatory cytokines, and a disturbed expression of key transcription factors, as observed in lesional plaques, suggesting a crucial importance of combining the pathological phenotype of cutaneous cells to T cells in order to generate a relevant model for psoriasis. Finally, we found this skin model to be responsive to methotrexate treatment, making it a valuable tool for drug development.

## 1. Introduction

Psoriasis is a T-cell mediated autoimmune disease associated with three main characteristics: Aberrant growth and hyperproliferation of epidermal keratinocytes, altered epithelial differentiation, and leukocyte infiltration, causing secretion of inflammatory mediators [1,2]. CD4-positive T cells infiltrate the dermis, while CD8-positive T cells migrate both within the dermal and epidermal compartments [3,4]. The proven clinical efficacy of drugs to inhibit or block the immune system, particularly lymphocyte-induced secretions, demonstrates the importance of T cells in the development and maintenance of the pathology [5,6,7,8]. 

Evidence of the importance of T cell-induced inflammatory cytokines is abundant. Already in 2004, Boyman et al. observed in a murine model deficient in type I and type II interferon receptors, and for recombination activating gene 2, that the activation of resident T cells, mediated by TNF-α, is necessary and sufficient for the development of psoriatic lesions [9]. Recently, van den Bogaard et al. developed a dermal equivalent model composed of a decellularized and deepidermized dermis, on which healthy keratinocytes are seeded, and activated CD4-positive cells are injected [10]. 

The authors demonstrated that the injection of these lymphocytes altered the epidermal differentiation towards an activated and inflammatory psoriatic-like phenotype. In vitro polarized lymphocytes, towards a Th1 and/or Th17 profile, would participate directly in the induction of inflammation, in contrast to Th2 cells [10], as already demonstrated on psoriasis skin lesions [11,12]. Our team has recently demonstrated that the supplementation of reconstructed lesional tissue culture media via a combination of IL-1α, TNF-α, IL-6 and IL-17A, leads to upregulation of genes known to be deregulated within in vivo lesional psoriatic skin (*S100A12*, *IL-8*, *DEFB4A*, *KYNU*) [13]. Another study has also shown that the addition of a cytokine cocktail (IL-1α, TNF-α, IL-6) leads to the expression of proteins associated with psoriasis such as SKALP, hBD-2, keratin-16, TNF-α and IL-8 [14]. Similarly, the grafting of a reconstructed human skin produced in vitro in a murine model, followed by the subsequent injection of in vitro polarized Th1 lymphocytes or a cocktail of IL-17 + IL-22 recombinant cytokines, combined with mild abrasive treatment, induced the development of a psoriasis-like phenotype in these mice [15]. The grafting of a complete reconstructed skin produced in vitro by tissue engineering from cells obtained from a non-lesional site on an immunodeficient SCID mouse, and then injected with autologous immunocytes, developed a characteristic appearance of the pathology, not observable with healthy cutaneous cells, again suggesting a crucial contribution of the immune system in the development of the pathology [16]. 

To date, too few three-dimensional organotypic culture models faithfully reproduce the cellular and molecular interactions between epithelial and immune cells. It is therefore essential in new models to pay particular attention to the immune component, in order to mimic the inflammatory tridimensional microenvironment. Incorporation of immunity fulfills the gap between irrelevant two-dimensional models of co-cultures and murine models, which do not recapitulate the human cutaneous structure, and complicate the understanding of the role of each cellular component in initiating or maintaining the pathology. Three-dimensional cell cultures are deemed superior to monolayers owing to increased extracellular matrix formation, cell-to-cell and cell-to-matrix interactions, very important for differentiation, proliferation and cellular functions in vivo. To develop new therapeutic agents targeting the immune system, we need the most complete model, combining key cells to predict the molecular exchanges between the involved cells. 

The aims of this study were (i) to develop and analyze the effect of activated T cells in lesional reconstructed skin models (LS + T), compared to T cell-free healthy (HS) and lesional (LS) skin models, (ii) to evaluate the impact of T cells on the lesional skin phenotype and on psoriatic key markers, and (iii) to validate the promising in vitro immunocompetent inflammatory model via an immunosuppressive pharmacological therapy. To meet these objectives, we sought to compare the protein expression profiles, secreted cytokines, and the response to current treatment between the different reconstructed tissues. Herein, we show that the immunocompetent lesional skin model is the only model to reflect all the key characteristics associated with psoriasis. Activated T cells contribute to the development and maintenance of psoriatic inflammation in our lesional tissue-engineered skin model.

## 2. Results

### 2.1. Generation of In Vitro Three-Dimensional Immunocompetent Human Models

**Figure 1:**
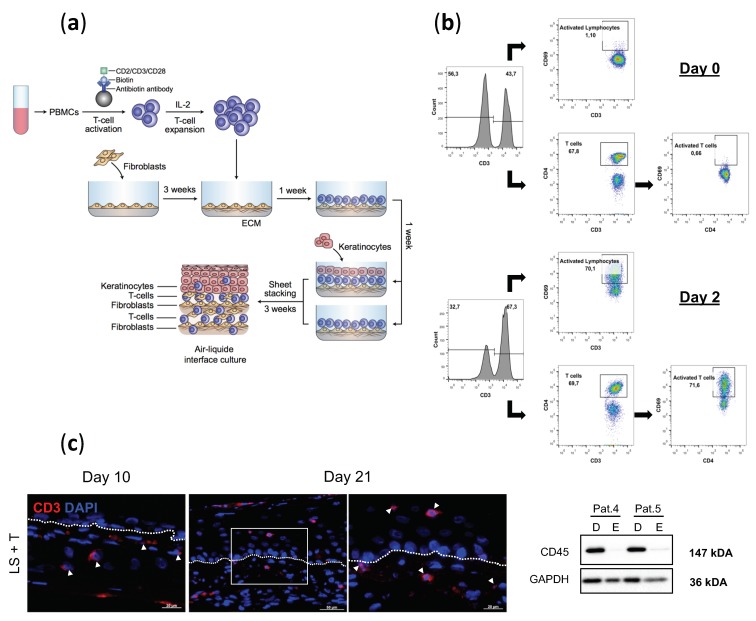
Generation of an immunocompetent skin model produced by tissue engineering. (**a**) Schematic experimental design for the generation of immunocompetent lesional (LS) tissue-engineered skin human model. (**b**) Flow cytometric quantification of CD4^+^ and CD69^+^ populations on day 0 and after 2 days of activation. Populations were analyzed for co-expression with CD3. Cells in the first panel were gated for viable cells. (**c**) Infiltration of T cells in LS (LS + T) was assessed by immunofluorescence staining for CD3 and DAPI at day 10 and 21 of the air-liquid culture. White arrows indicate positive CD3 cells. Dashed white lines represent the basement membrane. The third panel is a magnification of the delimited area of the second one. Scale bar (first and third panel) = 20µm. Scale bar (second panel) = 50µm. CD45 expression (relative to GAPDH) in the dermis (D) or epidermis (E) of LS reconstructed skin was evaluated by western blotting analysis. LS4 and LS5 refer to patients 4 and 5.

We developed an immunocompetent in vitro lesional skin model to mimic the infiltration of activated T cells observable in vivo in psoriatic lesion plaques. To this end, T cells isolated from peripheral blood mononuclear cells (PBMCs) of human healthy donors were activated in vitro and then added to the dermal sheets of lesional reconstructed skin models produced by tissue-engineering (Figure 1a). 

To verify MACSiBead activation of T cells, we determined by flow cytometry the level of CD69 expression, a common marker of T cell activation. The results indicated that the percentage of activated CD3^+^ CD69^+^ lymphocytes before the activation period (day 0) was 1.10%. CD4^+^ CD69^+^ T cells activated on day 0 represented a percentage of 0.66%. 

After 48-hour incubation of PBMCs in the presence of MACSiBead Particles loaded with biotinylated antibodies against human CD2/CD3/CD28, 70.1% of CD3^+^ lymphocytes co-expressed CD69, and 71.6% of CD4^+^ T cells also co-expressed CD69 (Figure 1b). In addition, the T cell activation kit promoted the almost complete depletion of CD14^+^ monocytes/macrophages, and thus positively isolated CD3^+^ T cells (Figure S1).

To evaluate the migratory potential of the lymphocytes, we stained T cell-infiltrated lesional tissues (LS + T) for CD3. The lymphocytes were present throughout the culture at the air-liquid interface, as they could be observed from day 10 to day 21. T cells migrated within the dermal compartment and towards the epidermis, as it was possible to visualize them on the other side of the dermo-epidermal junction at day 21 (Figure 1c). Western blot analysis of the leukocytes after mechanical separation of the dermis and the epidermis could be used to determine the proportion of lymphocytes infiltrated in the dermal compartment compared to the leukocyte number migrated into the epidermis. At day 21, most lymphocytes migrated within the dermal compartment, while few of them migrated to the epidermis, closely mimicking the in vivo infiltration of T cells in lesional plaques (Figure 1c). 

### 2.2. T Cells Infiltrated into the Dermis and the Epidermis Promoted Altered Differentiation of Keratinocytes 

**Figure 2:**
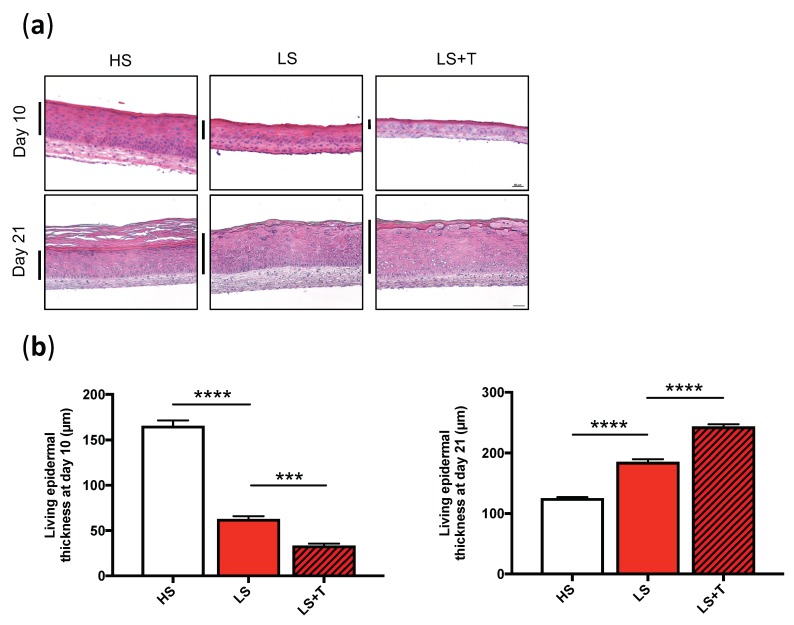
Migration of activated T cells within the dermis and the epidermis modified the turnover time of epidermal keratinocytes. (**a**) Histological analysis of reconstructed tissues at day 10 and day 21 of air-liquid culture. Black bars delimit the living epidermis of healthy (HS), lesional (LS), and lesional with T cells (LS + T) reconstructed skin. Scale bar = 50µm. (**b**) Quantification of the living epidermal thickness of healthy (HS), lesional (LS), and lesional with T cells (LS + T) reconstructed skin at day 10 (left panel) and 21 (right panel). The values are presented as mean ± SD (*n* = 3). Significant differences (***p < 0.001, ****p < 0.0001) are indicated by an asterisk.

To study the effect of T cell-addition on epidermal morphogenesis, we compared the thickness of the different tissues on day 10 and 21 of the air-liquid culture, as tissue culture during 21 days in air-liquid promotes and ensures a full differentiation and stratification of the epidermis. The thickness of lesional (LS) living epidermis was significantly lower than that of healthy skin (HS) at day 10 (LS: 63 ± 3µm, HS: 166 ± 6µm, *p* < 0.0001). However, the opposite was observed at day 21, where the LS epidermis was significantly thicker (LS: 186 ± 4µm, HS: 126 ± 1µm, *p* < 0.0001) (Figure 2a,b). In fact, while HS reached maximum thickness at day 10, the LS tripled the thickness of their epidermis between day 10 and day 21. Thus, the in vitro epidermal differentiation of LS was delayed compared to that of HS (Figure 2a,b). Interestingly, although the infiltration of activated T cells significantly reduced the thickness of LS at day 10 (LS: 63 ± 3 µm, LS + T: 34 ± 2 µm, *p* < 0.001), it drastically increased the thickness of LS at day 21 (LS: 186 ± 4 µm, LS + T cells: 244 ± 3 µm, *p* < 0.0001), suggesting hyperproliferation of lesional keratinocytes in the immunocompetent skin model, despite a delayed onset of epidermal differentiation (Figure 2a,b). 

### 2.3. Activated T Cells Induced Hyperproliferation of Lesional Keratinocytes 

**Figure 3:**
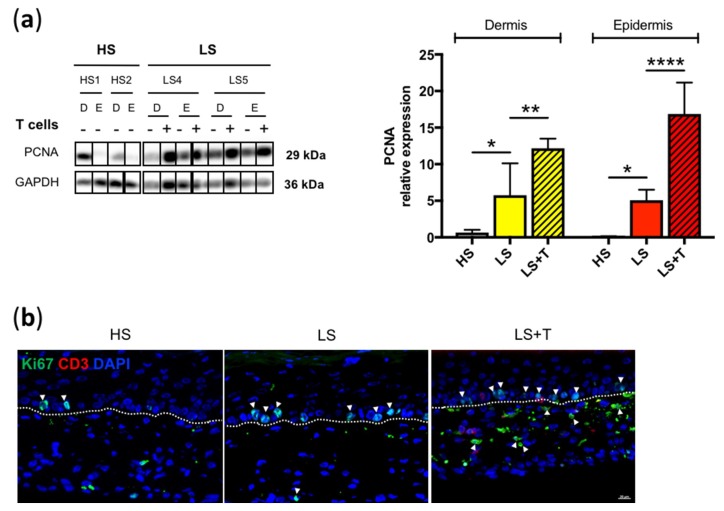
Impact of T cells on cell proliferation. (**a**) Western blot analysis and quantification of PCNA protein expression (relative to GAPDH) in the dermis (D) or the epidermis (E) of healthy (HS), lesional (LS), and lesional with T cells (LS + T) reconstructed tissues. The values are presented as mean ± SD (*n* = 2). Significant differences (*p < 0.05, **p < 0.01, ****p < 0.0001) are indicated by an asterisk. HS1 and HS2 refer to healthy patients 1 and 2. LS4 and LS5 refer to psoriatic patients 4 and 5. (**b**) Immunofluorescent staining of healthy (HS), lesional (LS), and lesional with T cells (LS + T) reconstructed skin, costained with Ki67, CD3, and DAPI. White arrows indicate positive Ki67 cells in the dermis and the epidermis. Dashed white lines represent the basement membrane. Scale bar = 20 µm.

To deeper investigate whether activated T cells affect these cells’ proliferation of skin models, we analyzed the basal expression level of proliferating cell nuclear antigen (PCNA), a common marker for the visualization of DNA replication in living cells. Mechanical separations between the dermis and the epidermis were performed on the different skin models, with or without activated T cells. The relative expression of PCNA was higher in the dermal and epidermal compartment of LS compared to the HS, where its expression was decreased within the two skin compartments. The addition of T cells potentiated the proliferation within the dermal compartment of lesional skin models, and even more in the epidermal compartment of LS (Figure 3a). Immunofluorescence analysis of the expression of the proliferation marker Ki67 demonstrated that T cell-free lesional reconstructed tissues had a higher proliferation rate of basal keratinocytes than that observed in HS, in agreement with observations made in vivo [17]. 

Moreover, the considerable increase in the proliferation of basal keratinocytes in reconstructed immunocompetent lesional tissues underlined the importance of activated T cells in the proliferation process (Figure 3b). It was also possible to observe an impact of leukocytes on the increase of dermal fibroblasts proliferation in the lesional immunocompetent skin model, consistent with the expression of PCNA by Western Blot (Figure 3a,b). PCNA marker can be expressed by activated T cells.

### 2.4. Infiltration of Lymphocytes in Reconstructed Lesional Skin Model Resulted in Increased Secretion of Proinflammatory Factors 

One of the main characteristics associated with psoriasis is the establishment of a chronic and sustained inflammatory response localized to the lesion plaques. Communication between the different cellular actors via the secretion of pro-inflammatory factors contributes to the development of the pathogenesis. Psoriasis is a Th1 and Th17 cell-mediated disease, and the presence of CD4^+^ Th1 cells is increased in lesional plaques, as well as the levels of cytokines consistent with the Th1 profile, such as IFN-γ and TNF-α [11]. Additionally, the number of Th17 cells and secretion of downstream effector molecules, such as IL-17A, IL-17F, and TNF-α, are clearly enhanced at the lesion [12,18,19]. 

**Figure 4:**
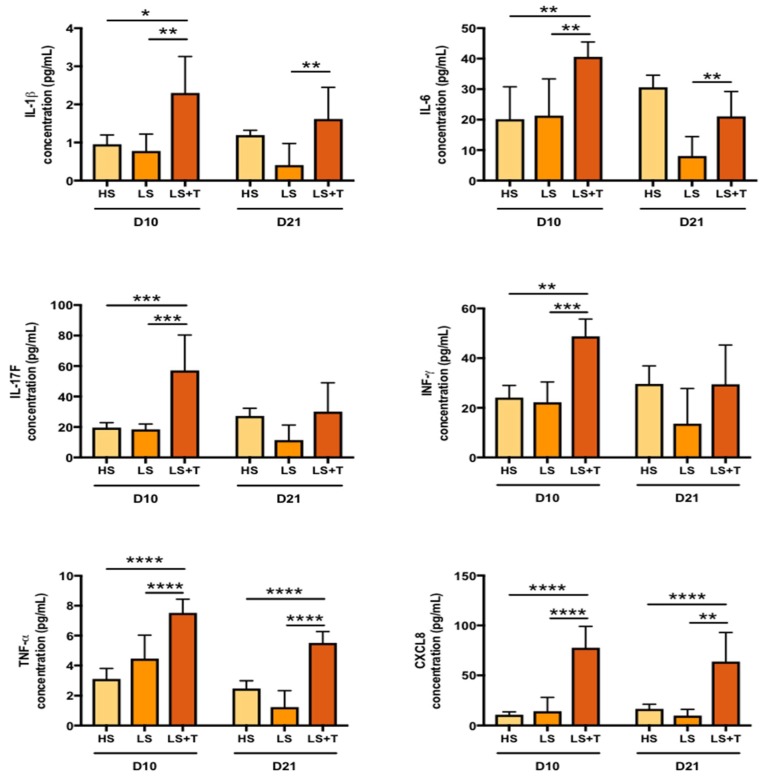
Increased secretion of proinflammatory factors generated by T cells in tissue-engineered lesional skin models. Analysis of protein expression of the culture supernatants of healthy (HS), lesional (LS), and lesional with T cells (LS + T) skin models at day 10 and 21 of the air-liquid culture. The values are presented as mean ± SD (*n* = 3). Significant differences (*p < 0.05, **p < 0.01, ***p < 0.001, ****p < 0.0001) are indicated by an asterisk.

We investigated whether our three-dimensional immunocompetent organotypic model was mimicking this inflammatory cascade at protein levels. The expression of IFN-γ, IL-1β, IL-17F, TNF-α, IL-6 and CXCL8 analyzed at day 10 was similar in LS and HS (Figure 4). Activated lymphocytes contributed directly or indirectly to the significant increase in the secretion of Th1 and Th17 mediators in lesional skin (LS + T) models compared to its control without activated T cells (LS) (Figure 4). Finally, IL-4 expression was not detected in tissues (data not shown). 

At day 21, secretion of pro-inflammatory factors was still visible, and the increase in expression of IL-1β, TNF-α, IL-6 and CXCL8 was still significant in LS + T compared to LS (Figure 4). Lesional immunocompetent skin models promoted sustained and chronic inflammation induced by T cells. 

### 2.5. T Cells Promoted the Activation of Signaling Pathways Involved in the Pathogenesis of Psoriasis

Modeling psoriatic inflammation implied the mimicry of the signaling pathways involved in the development and maintenance of inflammation. It is known that some cytokines, such as IFN-γ and TNF-α, activate intracellular transcription pathways, such as signal transducer and activator of transcription 1 (STAT1), and STAT3, which regulate the expression of many molecules involved in the inflammatory response [20,21,22]. Also, gene expression of *STAT1* was shown to be increased in psoriatic skin [23]. For this reason, we evaluated the expression of STAT1 and their activation by phosphorylation after T cell infiltration into lesional tissues.

**Figure 5:**
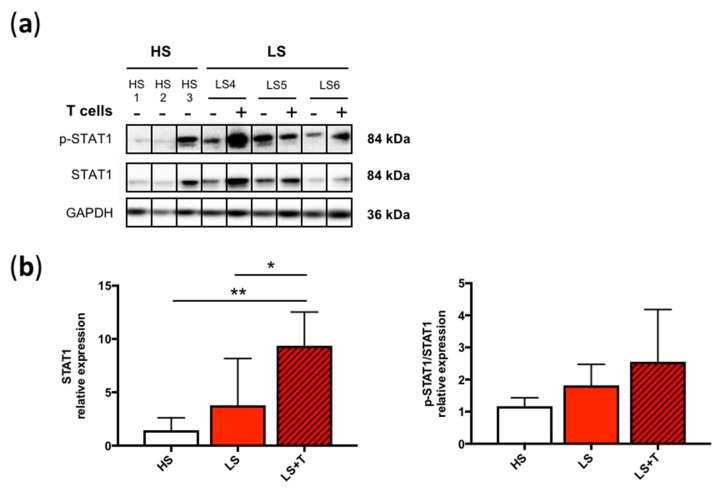
T cells induced immune-dependent STAT1 signaling in tissue-engineered skin models. (**a**) Western Blot and (**b**) quantification of STAT1 (relative to GAPDH), and phospho-STAT1 (p-STAT1)/STAT1 in healthy (HS), lesional (LS) and lesional with T cells (LS + T) tissue-engineered skin models. Significant differences (*p < 0.05, **p < 0.01, ****p < 0.0001) are indicated by an asterisk. Error bars represent SD (*n* = 3). HS1, HS2, and HS3 refer to healthy patients 1, 2, and 3. LS4, LS5, and LS6 refer to psoriatic patients 4, 5, and 6.

The baseline levels of STAT1 were not significantly different in HS, and LS models, although these levels tend to be increased in LS (Figure 5a,b). Activated T cells significantly increased the expression of STAT1 in lesional reconstructed tissues. Surprisingly, activation of STAT1, highlighted by the ratio p-STAT1/STAT1, was only slightly increased in LS + T. (Figure 5a,b). These results indicated that T cells are partly responsible for the activation of the STAT1 pathway.

### 2.6. A Methotrexate Treatment Improved the Epithelial Phenotype and Decreased T-Cell Induced Inflammation

To pharmacologically validate the developed models, we investigated whether our models reflected the anti-inflammatory and anti-proliferative effect of methotrexate. Methotrexate (MTX) is widely used as a first-line treatment in moderate to severe psoriasis. It is considered as an antimetabolite due to its antagonistic effect on folic acid metabolism. TNF production by T cells is an important target of methotrexate, as it reduces TNF serum levels in vivo. Methotrexate has also shown anti-proliferative effect on keratinocytes [24]. 

**Figure 6:**
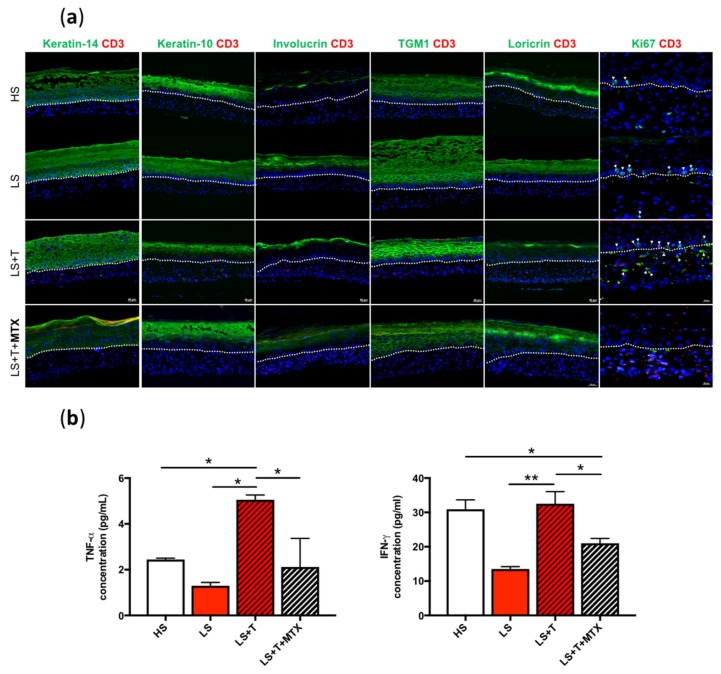
Alleviation of the epithelial phenotype and decrease in the production of TNF-α and IFN-γ after methotrexate treatment. (**a**) Expression of keratin-14, keratin-10, involucrin, transglutaminase 1 (TGM1), loricrin, and Ki67 (costained with DAPI) monitored by immunofluorescence in healthy (HS), lesional (LS), and immunocompetent lesional skin models treated (LS + T + MTX) or not (LS + T) with methotrexate (MTX). Dashed white lines represent the basement membrane. Scale bar = 50 µm. (**b**) Protein expression analysis of the culture supernatants of healthy (HS), lesional (LS), and lesional with T cells (LS + T) skin models, with (LS + T + MTX) or without (LS + T) methotrexate treatment, at day 21 of the air-liquid culture, assessed with Luminex xMap technology. The values are presented as mean ± SD (*n* = 3). Significant differences (*p < 0.05, **p < 0.01) are indicated by an asterisk.

The passage of keratinocytes from the basal layer to the spinous layer is characterized by a change in the expression of keratin from the basal layer, such as keratin-14, to that of suprabasal epidermal keratin, such as keratin-10. The expression of keratin-14 was increased in LS compared to HS. The migration of T cells into the dermal compartment of LS contributed to the increase in the expression level of keratin-14 (Figure 6a). The synthesis of keratin-14 was reduced in LS + T treated with methotrexate (Figure 6a). 

The infiltration of T cells considerably reduced the synthesis of keratin-10 in lesional tissues (Figure 6a), as observed in vivo [25,26]. After treatment with methotrexate, keratin-10 was synthesized in all the suprabasal layers of the lesional tissues (Figure 6a).

Early differentiation markers such as involucrin and transglutaminase 1 are known to be upregulated in lesional skin, namely in the spinous and granular layers, compared to healthy skin [27,28]. These proteins were indeed overexpressed in LS while they were weakly expressed in HS, as observed in vivo. The addition of CD3-positive cells led to a significant increase in the expression of involucrin in LS + T (Figure 6a). In addition, activated T cells promoted the overexpression of transglutaminase 1 in LS + T (Figure 6a). Treatment with methotrexate normalized the expression of involucrin and transglutaminase 1. 

Loricrin is a marker of late differentiation, and its expression is diminished in psoriatic lesions. As observed in vivo, loricrin synthesis was localized in the granular layer of the HS epidermis, and its expression was greatly decreased in LS. The infiltration of T cells considerably reduced the synthesis of loricrin in LS + T, since its expression was now limited to one epidermal layer (Figure 6a). Finally, it was interesting to note that loricrin, absent in LS, was finally expressed following treatment with methotrexate (Figure 6a). 

In addition, treatment with methotrexate reduced the proliferation of epidermal basal keratinocytes in LS + T (Figure 6a). The secretion of proinflammatory cytokines IFN-γ and TNF-α was significantly decreased following a 7-day treatment with methotrexate in LS + T, compared to untreated LS + T (Figure 6b). 

Here, we showed that T cells induce an alteration of the epithelial differentiation process and that methotrexate attenuated the activated phenotype induced by T cells in lesional tissues. This reinforced the validity of our model, which effectively responded to an immunosuppressive treatment widely used to treat psoriasis. 

## 3. Discussion

In this study, we clearly demonstrate that skin models reconstructed with lesional psoriatic cells displayed a strong psoriasis-like activated inflammatory phenotype after infiltration of activated T lymphocytes into an in vitro 3D tissue microenvironment. Indeed, these skin models exhibited a hyperplastic epidermis (acanthosis), altered differentiation of keratinocytes, hyperproliferation of basal keratinocytes, and the increased secretion of pro-inflammatory cytokines. This study also demonstrates that psoriatic keratinocytes show in vitro a delayed onset of epidermal differentiation, as already demonstrated by Bernerd et al. in 1992 [26], highlighting the abnormality of the basal layer of the epidermis, in agreement with alterations of keratin expression in our lesional model. Yet, once the epidermis has been stratified, the keratinization process seems to be accelerated in lesional models compared to healthy models, in agreement with the observations in vivo [29]. 

In addition, the moderate activation of STAT1 signaling pathways emphasizes the relevance of our model, although a stronger increase was expected. These results were consistent with the in vivo observations: The increase of the transcription factors expression (STAT1 and STAT3) and their subsequent activation triggered signaling pathways involved in the initiation of the inflammation and its maintenance, leading to an increase in basal keratinocyte proliferation and further altering their process of differentiation, thereby forming a positive feedback. Moreover, this new model responded to an immunosuppressive treatment widely used to treat psoriasis, thus reinforcing its validity. 

To our knowledge, this is the first developmental research article of an in vitro cutaneous immunocompetent model to analyze the impact of activated T cells on the development of psoriasis from inflammation in tissue-engineered skin models generated with lesional skin cells. In the model developed by van den Bogaard et al. [10] where CD4-positive T cells were injected into healthy reconstructed skin, the experimental conditions did not appear to induce hyperproliferation of basal keratinocytes, since the gene expression of the *Ki67* proliferation marker was not increased after injection of the CD4-positive T cells. Our model of in vitro immunocompetent lesional skin displays hyperproliferation of epidermal keratinocytes. PCNA markers can be expressed by activated T cells [30]. However, Figure 1 demonstrates that activated T cells remain predominantly in the dermal compartment, and only a minority pass through the dermal-epidermal junction. Thus, the increase of PCNA expression in the epidermal compartment of lesional skin can be incriminated to keratinocytes. Similarly, there was no colocalization between Ki67 and CD3 in the dermal compartment of lesional skin with activated T cells, confirming the role of T cells in the hyperproliferation of keratinocytes and fibroblasts. Taken together, these results suggest that the pathological character of epithelial and dermal cells is necessary and essential to trigger epidermal hyperproliferation. These observations emphasized the importance of the initial pathological phenotype of lesional cells which would be able to keep in memory their abnormal state. This pathological memory would lead to a stronger and faster secondary response to the immune stimulus. The pathological memory was also reported in other studies, which found that IL-17 producing T cells, sensitized to imiquimod, were shown to travel to distant skin sites and persist for several months. These memory-like cells, under a second stimulation, spread more quickly and produce more IL-17, leading to a greater inflammatory response to the skin than when first exposed to the sensitizer [31]. Another study by Hartwig et al. confirms this hypothesis, since the authors demonstrated that γδT cells producing IL-17 trigger a stronger and faster response after a second stimulation with Aldara cream, since these cells persist in the dermis [32]. It could be envisaged that a similar secondary response is generated in our lesional immunocompetent model, as evidenced by the increase expression of proinflammatory factors (IFN-γ, IL-1β, IL-17F, TNF-α, IL-6 and CXCL8), compared to T cell-free lesional skin models (Figure 4). Our results also underlined the crucial role of activated T cells in the psoriasis-like modification of the in vitro epidermal differentiation process.

It was shown in this study that the incorporation of the T cell increased the secretion of pro-inflammatory cytokines at both day 10 and 21. However the levels of cytokines secreted at day 21 in LS + T did not remain significantly different from those of HS, as observed at day 10. Several hypotheses can be stipulated as to the decrease in the secretion of proinflammatory cytokines between day 10 and day 21 in lesional skins. The first is that T cells could get functionally impaired or exhausted. It has already been shown that T cells upon long-term exposure to tumor antigen can adopt an exhausted phenotype [33,34,35]. In addition, chronic infections can cause a weakening response and a shutdown of cell-mediated immunity. A second option could be the adaptation of T cells to chronic inflammation conditions. For instance, there is some evidence that T cells can adopt a specialized phenotype in some conditions of chronic infections, via a memory-like T cell formation that transmits an exhausted phenotype to other short-lived effector T cells [35]. Further analyses are required to confirm or not these hypotheses in the pathogenesis of psoriasis. Another hypothesis is that the prolonged culture of keratinocytes causes a decrease in the synthesis of pro-inflammatory cytokines, visible by the decrease in these secretions between day 10 and day 21 in lesional skins (LS).

However, the integration of activated T cells in lesional skin (LS + T) compensates for this decrease in secretion, and therefore maintain a sustained level of inflammation in lesional skins, at least until day 21 of culture at the air-liquid interface, since the basal levels of all cytokines are higher than those of lesional skins (LS).

At the lesional site, the recruitment of particular lymphocytic subpopulations and the induction of a specific Th1/Th17 response results from precise secretion of a panel of cytokines by antigen presenting cells, dendritic cells, previously activated by various stimuli [36,37]. Despite the absence of antigen presenting cells, our study suggested that our skin cells directly influence and drive the polarization of activated T cells within tissues. Indeed, IL-4, the signature cytokine of Th2 lymphocytes, was not detected in our culture supernatants, whereas Th1 cell-specific IFN-γ cytokine, IL-17F, and TNF-α were secreted, demonstrating an orientation of T cells towards a Th17 profile. Immunofluorescence or cytometric analysis of Th1 and/or Th17 cell markers on day 21 of air-liquid culture would allow us to identify the lymphocyte subpopulations present in our models, and deduce the influence of lesional skin cells on the polarization of T cells in vitro. 

Surprisingly, IL-17A was not detected in our culture supernatants, even after infiltration of T cells into lesional tissues. A study published by Cheuk et al. indicated that epidermal CD8 T-cells from resolved plaques mainly produced IL-17A, which drives the production of pro-inflammatory cytokines and chemokines by keratinocytes [38]. In contrast, IL-17F would be produced by CD4-positive T cells, and would participate in the development of psoriatic inflammation via an induction of IL-6 by keratinocytes [39]. We suggest that our T cell activation system promoted the presence of CD4^+^ T cells and depleted CD8^+^ T cells. Besides, it would be interesting to enrich this new model with either CD4^+^ T cells, or CD8^+^ T cells, or a mix of both to understand the exact role of these two subpopulations in the development and the chronicization of psoriasiform inflammation. Interestingly, the activation of STAT1 was not evident in lesional skin models. Shi et al. demonstrated that IL-17A regulated the expression of keratin-17 via STAT1 and STAT3-dependent mechanisms [22]. If IL-17A is not secreted, as presumably in the absence of the protein detection in culture supernatants, the activation of this signaling pathway could be limited. 

Our study has shown that a treatment with methotrexate of lesional immunocompetent skin models led to a reduction in the secretion of pro-inflammatory cytokines (IFN-γ and TNF-α). We also demonstrate that methotrexate counteracted T cell-mediated effects in our tissues, by decreasing basal keratinocyte proliferation, and restoring a healthy epithelial phenotype associated with normal differentiation of epidermal keratinocytes. Our team has already demonstrated that treatment with retinoic acid of lesional skin models produced without T cells, improved the differentiation of epidermal keratinocytes and decreased the thickness of the living epidermis [40]. Thus, retinoic acid could play a role on the non-immune component associated with psoriasis. A combination of these two agents, methotrexate and retinoic acid, might be worth studying, since each drug could act on a different pathway.

Herein, we developed a unique complete model representative of cellular and molecular interactions in psoriatic lesional plaques. However, we are aware that the addition of dendritic cells to our model, for instance, would highlight the activation of T cells by these antigen presenting cells. Nestle et al. showed that plasmacytoid predendritic cells accumulated in the skin of psoriatic patients, and produced IFN-α early during disease development, promoting the activation and expansion of T cells, leading to the development of psoriasis [41]. In 2008, Albanesi et al. demonstrated that accumulation of plasmacytoid dendritic cells (pDCs) is associated almost exclusively with the concomitant presence of neutrophils and mast cells in the dermis of psoriatic lesional skin, whereas the presence of neutrophils in the psoriatic skin epidermis is associated with a limited presence of pDCs [42]. These results emphasize the specific importance of each cell type in the different phases of the disease. Also, the addition of a nerve component could be interesting as an enhanced release of neuropeptides is found in psoriasis [43,44]. Similarly, generating a completely autologous model to mimic the pathology of psoriasis requires taking both a skin biopsy and a blood sample from the same donor. This model represents a new and innovative challenge, and would limit the bias induced by allogeneic cells.

In this study, we have demonstrated that the development of a relevant psoriatic skin model both requires an immune component, essential for the development and maintenance of inflammation, and pathological skin cells, resulting from psoriatic lesions. The combination of psoriatic cells, both fibroblasts and keratinocytes, and activated T cells effectively mimics psoriatic inflammation and reflects major histopathological features associated with the pathology. This unique and advanced preclinical model could be used to test new potential immunosuppressive drugs for the treatment of psoriasis.

## 4. Materials and Methods 

### 4.1. Patients and Biopsies

The subjects were all Caucasian females and males. Three healthy patients aged between 18 and 46 years, and three patients with plaque psoriasis aged between 46 and 64, were recruited. Six-millimeter punch biopsies were taken from healthy (normal skin from a non-psoriatic patient) and lesional skin (from a psoriatic patient). Psoriatic patients received the same treatment prior to biopsy (methotrexate) and all have plaque psoriasis (between 10 to 20% of body surface area affected). This study was conducted in agreement with the Helsinki declaration, and performed under the guidelines of the Research Ethics Committee of the CHU de Québec. All patients were given adequate information to provide written consent.

### 4.2. T-Cell Isolation and Stimulation

Mononuclear cells were isolated from peripheral blood obtained from six healthy donors by Ficoll-Hypaque density gradient centrifugation, followed by three periods of adhesion (1 h, 2 h and overnight) on a plastic support to isolate the adherent monocytes / macrophages from the remaining T cells in suspension. All patients, different from those whose skin cells were extracted, were given adequate information to provide written consent. T cells were then activated with anti-biotin MACSiBead Particles loaded with biotinylated antibodies (against human CD2/CD3/CD28) using the T cell activation/expansion kit from Miltenyi Biotech GmbH (CA, USA). Anti-Biotin MACSiBead Particles loaded with the biotinylated antibodies are used to mimic antigen-presenting cells and activate T cells from PBMCs. Briefly, loaded anti-biotin MACSiBead™ were added to PBMCs at a T-cell/bead ratio of 1:2, and incubated for 2 days at 37 °C, and 8% CO_2_, according to the manufacturer’s instructions (Miltenyi Biotech GmbH, CA, USA). After removal of the beads by a magnetic device, these T cells were washed and added to the dermis compartment of lesional reconstructed skin models. 

### 4.3. In Vitro Reconstructed Skin

In vitro reconstructed skin models have been produced according to the self-assembly method, with some minor modifications [45]. Each reconstructed skin contains fibroblasts and keratinocytes of the same donor, and therefore of the same age and same sex. Briefly, 0.08 × 10^6^ human dermal fibroblasts were seeded in 12-well plates, and cultured with ascorbic acid at a concentration of 50 mg/mL (Sigma-Aldrich, MO, USA). The dermal tissues were enabled to produce their own extracellular matrix for three weeks at 37 °C and 8% CO_2_. After this time frame, activated T cells (0.4 × 10^6^ per sheet) were seeded on dermal fibroblast sheets for 1 more week, in presence of 30 U/mL recombinant human IL-2 (R&D Systems, MN, USA). Then, human keratinocytes (0.75 × 10^6^) were seeded on the top of one dermal immunocompetent sheet, and cultured in DMEM-HAM with ascorbic acid and IL-2 submerged for 7 days, allowing the keratinocytes to proliferate. Two sheets were then stacked and raised at the air-liquid interface, allowing the cells to differentiate. Cell culture media were changed three times a week. Cell culture supernatants were taken at day 10 and day 21 during the air-liquid culture. Healthy and lesional skin models without activated T cells served as controls.

### 4.4. Methotrexate Treatment 

Lesional skin models with activated T cells were treated with methotrexate (methotrexate injectable USP, 50 mg/2 mL, IL, USA) within the last week of culture at the air-liquid interface. To administer a dose of 20 mg per week, we added directly to the fresh culture medium, three times a week, 734 μM of solution for injection.

### 4.5. Flow Cytometry

Cells from human blood were labelled at day 0 after Ficoll-Hypaque density gradient centrifugation and at day 2, after stimulation with loaded MACSiBead, with the following antibodies: CD3-APC, CD4-PE (both BD Biosciences, CA, USA), CD69-FITC (Biolegend, CA, USA) and CD14-PE (ImmunoTools, Berlin, Germany). Labeled cells were sorted on a BD FACSMelody cell sorter. Cells were first gated for viability using the 7-AAD stain. 

### 4.6. Histology and Immunofluorescence Stainings

For living epidermal thickness and morphology analyses, deparaffinized 5-µm tissue sections were dipped in running tap water before incubation with hematoxylin and eosin. Tissues were then dehydrated, and stained slides were mounted in a toluene-based mounting medium (Thermo Fisher Scientific, MA, USA). For immunofluorescence staining, tissues were embedded in O.C.T (Sakura Finetek, USA) and 6-µm cryosections were fixed in acetone before processing. Samples were then incubated in 1% bovine serum albumin in PBS overnight at 4 °C with primary antibodies (Table S1). The next day, tissues were incubated with Alexa Fluor 488 donkey anti-rabbit IgG (H + L) and/or Alexa Fluor 594 goat anti-mouse IgG (H + L) (both Life Technologies, CA, USA, 1:1200) for 45 minutes at room temperature in a dark humidified chamber. Nuclear counterstaining using DAPI Fluoromount-G (SouthernBiotech, AL, USA) was carried out routinely. Samples were observed using a Zeiss Axio Imager M2 microscope equipped with an AxioCam ICc1 camera or AxioCam HR Rev3 camera (Oberkochen, Allemagne). 

### 4.7. Protein Extraction and Western Blot Analysis

If necessary, the dermis was separated from the epidermis manually using forceps. After cryogenic grinding, tissues were lysed with RIPA buffer containing a protease inhibitor cocktail (Roche, Bâle, Switzerland). Protein extracts (15µg) were then loaded onto a 12% reducing SDS-PAGE gel. After transfer on a nitrocellulose membrane (Bio-Rad, CA, USA), and blocking in TBS containing 0.1% Tween-10 and 5% nonfat milk for 1 hour, blots were incubated overnight at 4 °C with mouse primary antibodies: PCNA (Biolegend, CA, USA, 1:1000), or with rabbit primary antibodies: CD45 (Abcam, Cambridge, England, 1:1000), STAT1, and p-STAT1, (all Cell Signaling Technology, MA, USA, 1:1000), or with a goat anti-GAPDH (Bio-Rad, CA, USA, 1:20,000). Secondary antibodies used were 1:2500 dilution of goat anti-rabbit HRP labeled, goat anti-mouse HRP labeled (both Thermo Fisher Scientific, MA, USA), and rabbit anti-goat HRP labeled (Novex by Life Technologies, CA, USA). The proteins of interest were detected using the ECL Prime Western Blotting Detection Reagent (GE Healthcare, Little Chalfont, UK). Quantification of immunoblots were performed by densitometry in ImageJ. 

### 4.8. Cytokine Production Quantification by ELISA and Luminex

Culture media were collected during the air-liquid culture (on days 10 and 21), centrifuged and stored at −80 °C until used. Secretion of cytokines and chemokines was evaluated by using a multiplex immunoassay from Bio-Rad (Bio-Plex Pro Human Th17 cytokine panel 15-plex, Bio-Rad, CA, USA). 15 soluble proteins involved in the Th17 immune response pathway (IL-1β, IL-4, IL-6, IL-10, IL-17A, IL-17F, IL-21, IL-22, IL-23, IL-25, IL-31, IL-33, IFN-γ, sCD40L, TNF-α) were measured on a 96-well plate, using the Luminex xMAP technology. CXCL8 was quantified by using an ELISA kit purchased from R&D Systems (MN, USA). All measurements were performed in duplicate.

### 4.9. Statistical Analysis

Data are shown as mean ± SD and represent three independent experiments. Statistical analyses were performed using the one-way Analysis of Variance (ANOVA) test, followed by post hoc analysis using Tukey’s multiple comparison tests, and a two-way ANOVA test followed by post hoc analysis using Tukey’s multiple comparison tests for Luminex experiments. Values of *p* < 0.05 were considered significant. 

## Supplementary Materials

Supplementary materials can be found at https://www.mdpi.com/1422-0067/20/7/1670/s1.
