# Supplementary material for: Infiltration of T Cells into a Three-Dimensional Psoriatic Skin Model Mimics Pathological Key Features
Source: Int J Mol Sci. 2019 Apr 3;20(7):1670. doi: 10.3390/ijms20071670 (PMC6479293; doi:10.3390/ijms20071670)
Supplement: Supplementary file 1 [file ijms-20-01670-s001.pdf]

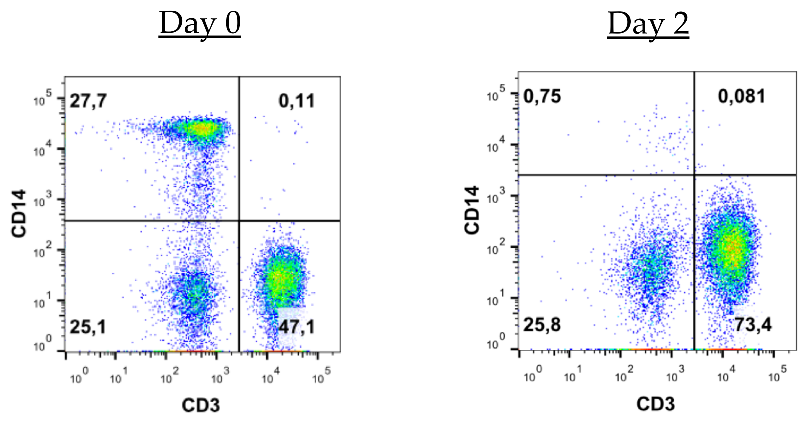

Figure S1. Flow cytometric quantification of $CD14^{+}$ and $CD3^{+}$ populations on day 0 and after 2 days of T cell activation. Cells were gated for viable cells.

Table S1. Primary antibodies used for immunofluorescence analysis.

Antibodies	Host	Company	Dilution factor
CD3 (F7.2.38)	Mouse	Dako, CA, USA	1:5
Keratin-14	Rabbit	Biolegend, CA, USA	1:1600
Keratin-10	Rabbit	Sigma, MO, USA,	1:100
Ki-67	Rabbit	Abcam, Cambridge, England	1:400
Involucrin	Rabbit	Abcam, Cambridge, England	1:600
Loricrin	Rabbit	Proteintech, IL, USA	1:1000
Transglutaminase 1	Rabbit	Proteintech, IL, USA	1:1600
